# Brucine Sulfate, a Novel Bacteriostatic Agent in 3D Printed Bone Scaffold Systems

**DOI:** 10.3390/polym16101428

**Published:** 2024-05-17

**Authors:** Jinying Li, Shi Hu, Pei Feng, Yang Xia, Zihan Pei, Jiaxuan Tian, Kun Jiang, Liang Liu, Xiong Cai, Ping Wu

**Affiliations:** 1 Institute of Innovation and Applied Research in Chinese Medicine, Hunan University of Chinese Medicine, Changsha 410208, China; lijinying@stu.hnucm.edu.cn (J.L.); 2014600924@smail.xtu.edu.cn (Y.X.); peizihan@stu.hnucm.edu.cn (Z.P.); tianjiaxuan@stu.hnucm.edu.cn (J.T.); jk@stu.hnucm.edu.cn (K.J.); 2 State Key Laboratory of Precision Manufacturing for Extreme Service Performance, College of Mechanical and Electrical Engineering, Central South University, Changsha 410083, China; shi.hu@siat.ac.cn (S.H.); fengpei@csu.edu.cn (P.F.); 3 Institute of Biomedical and Health Engineering, Shenzhen Institute of Advanced Technology, Chinese Academy of Sciences, Shenzhen 518055, China; 4 Changde First Chinese Medicine Hospital, Changde 415000, China; 5 State Key Laboratory of Traditional Chinese Medicine Syndrome, Guangdong Provincial Hospital of Chinese Medicine, Guangdong Provincial Academy of Chinese Medical Sciences, The Second Affiliated Hospital of Guangzhou University of Chinese Medicine, Guangzhou 510006, China

**Keywords:** BS, drug-loaded bone scaffold, antibacterial activity

## Abstract

Bacterial infection is a common complication in bone defect surgery, in which infection by clinically resistant bacteria has been a challenge for the medical community. Given this emerging problem, the discovery of novel natural-type inhibitors of drug-resistant bacteria has become imperative. Brucine, present in the traditional Chinese herb *Strychnine semen*, is reported to exert analgesic and anti-inflammatory effects. Brucine’s clinical application was limited because of its water solubility. We extracted high-purity BS by employing reflux extraction and crystallization, greatly improved its solubility, and evaluated its antimicrobial activity against *E. coli* and *S. aureus*. Importantly, we found that BS inhibited the drug-resistant strains significantly better than standard strains and achieved sterilization by disrupting the bacterial cell wall. Considering the safety concerns associated with the narrow therapeutic window of BS, a 3D BS-PLLA/PGA bone scaffold system was constructed with SLS technology and tested for its performance, bacteriostatic behaviors, and biocompatibility. The results have shown that the drug-loaded bone scaffolds had not only long-term, slow-controlled release with good cytocompatibility but also demonstrated significant antimicrobial activity in antimicrobial testing. The above results indicated that BS may be a potential drug candidate for the treatment of antibiotic-resistant bacterial infections and that scaffolds with enhanced antibacterial activity and mechanical properties may have potential applications in bone tissue engineering.

## 1. Introduction

Bone defects occur in tens of millions of patients with severe bone trauma, poorly treated fractures, and bone tumors, and their clinical management continues to be a huge challenge [1,2,3]. With the development of tissue engineering techniques in recent years, the implantation of scaffolds or bone substitutes has become a new means of treating bone defects [4]. It is noteworthy that severe trauma could cause the body’s stress response, which results in low immunity in the organism [5]. The surgical procedure of scaffold implantation is prone to bacterial infections, potentially leading to bone graft failure [6,7]. Postoperative infections are commonly nosocomial and are mostly associated with nosocomial-resistant bacteria. Its treatment is a complex and convoluted process that requires the resolution of two major challenges: controlling the bone infection and repairing the bone defect [8]. Traditional treatment methods include irrigation, debridement, drainage, and so on, which have a high recurrence rate in the late stages of treatment due to their long treatment times and difficulty of care [9]. Therefore, the combination of antibiotics and bone scaffolding materials has become a new therapeutic approach [10]. Antibiotics were commonly used to treat infectious diseases, such as azithromycin, amoxicillin, and ofloxacin [11]. Due to antibiotic abuse, antibiotic resistance has become a growing health problem [12]. Once a patient developed antibiotic resistance, it would become more difficult to treat the infection. Some patients would suffer amputation or even death due to poor infection control [13].

Treatment options are limited by bacterial resistance, making it difficult to properly administer conventional anti-infective therapy [14]. In recent years, several new antimicrobial drugs have been on the market or in clinical trials [15]. Most of them are semi-synthetic antibiotics or fully synthetic antimicrobial drugs with known structural types [16]. As a preventive and therapeutic measure, the exploration and development of novel drugs derived from natural plants has become a hotspot in the fight against bacterial resistance due to their unique antibacterial activity and fewer adverse effects [17]. *Semen strychnine* is a mature seed of *Strychnos nux-vomica* L. *(Loganiaceae)* growing in tropical Asia. In recent years, the pharmacological effects of *Semen strychnine* have been studied more and more extensively. The extracts of *Semen strychnine* have been proven to have antimicrobial activity against *Bacillus subtilis* and *Salmonella* [18,19]. The total alkaloids of *Semen Strychnine* (TASS) are the main active ingredient of strychnine, which has antibacterial, anti-inflammatory, and analgesic effects [20,21]. Our previous studies have shown that bacterial growth is inhibited by the TASS by interfering with the synthesis of peptidoglycan and proteins and hindering the production of structural proteins and bacterial enzymes [22]. However, the specific components of TASS with antimicrobial activity have remained unknown. Brucine, an important active ingredient of the *Semen strychnine* extracts, was notable for its poor solubility, short therapeutic window, and toxic side effects [23].

The main three-dimensional (3D) printing techniques that have been studied are the following: Binder Jetting (BJ), Fused Deposition Modeling (FDM), Semi-solid Extrusion (SSE), Stereolithography (SLA), and Selective Laser Sintering (SLS) [24]. The SLS technology has a high degree of safety and does not require the use of organic solvents, making it capable of printing different substances on the same scaffold. It was frequently used to prepare composite scaffolds with good biocompatibility, biodegradability, and osteo-inductivity [25]. Thus, we considered constructing a 3D bone scaffold composite system using SLS technology. l-lactide (Poly(l-lactide), PLLA) has good biodegradability, and can be degraded to carbon dioxide and water, which are eventually absorbed by the human body [26,27]. Polyhydroxy acetic acid (Polyglycolide, PGA) is one of the most important polymer materials used in medicine because of its superior hydrophilicity [28,29]. Therefore, the blending of PLLA with PGA is expected to achieve good biocompatibility, biodegradability, and mechanical properties. BS drug particles were loaded into the blend of PLLA/PGA to give it good antimicrobial properties and biocompatibility and allow a slow and controlled release of BS to reduce its cytotoxicity [30,31].

In this study, BS was successfully prepared, and its purity was examined. In addition, a 3D-printed BS-loaded PLLA/PGA scaffold was constructed, and its morphological characterization was tested. The controlled release behavior of the scaffold was studied by high-performance liquid chromatography (HPLC), and the antimicrobial properties were investigated. Meanwhile, the antimicrobial mechanism of the drug-loaded bone scaffolds was explored, and their cytocompatibility, including cell adhesion and viability, was evaluated. Ideally, BS could be used as a plant-derived antimicrobial active substance in bone tissue engineering to exert local antimicrobial effects with the aim of improving the success rate of bone grafting surgery.

## 2. Experimental

### 2.1. Materials and Preparation

*Semen strychnine* was purchased from Hunan Zhenxing pharmaceutical Co., Ltd. (Huaihua, China) and was identified as dry and mature seeds of *Strychnos nux-vomica* L. *(Loganiaceae)* belonging to the family Loganiaceae by Dr. Zhong Li (professor in the Department of Identification of Chinese Medicine, Guangdong Pharmaceutical University). Brucine standards were purchased from the National Institutes for Food and Drug Control, China, and the purity of brucine standards was 95.9% (batch number: 110706-200505), respectively. PLLA powder, which has a molecular weight of 150 kDa and a melting point of 175 °C, PGA powder, which has a molecular weight of 100 kDa and a melting point of 224 °C, Both of the PLLA and PGA powders were obtained from Shenzhen Polymtek Biomaterial Co., Ltd. (Shenzhen, China) for use as biomaterials in 3D printing with SLS. *Escherichia coli* (*E. coli*, ATCC 25922) and *Staphylococcus aureus* (*S. aureus*, ATCC 6538P) were used as standard strains. Antibiotic-resistant strains of *E. coli* (201905489, resistant to gentamicin and ofloxacin) and *S. aureus* (20190509472, resistant to penicillin and methicillin) were isolated by Pro. Canrong Wu from the Microorganism Department at the Hunan University of Chinese Medicine and identified by microorganism tests and biochemistry identification.

**Figure 1:**
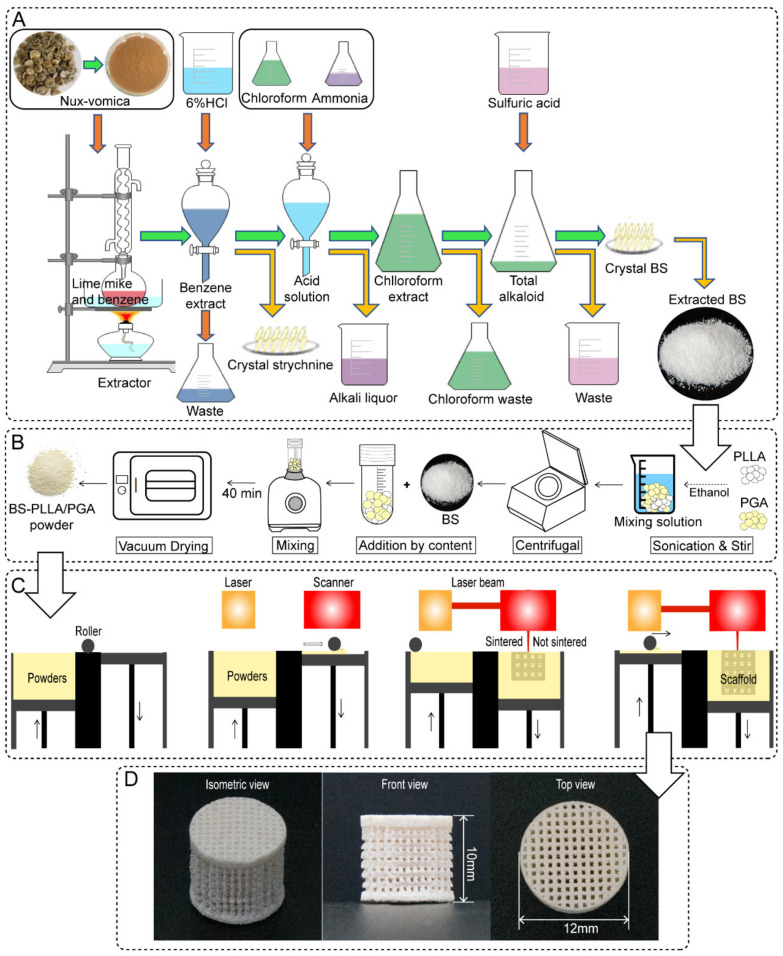
(**A**) The fabrication process of BS; (**B**) preparation of BS-PLLA/PGA drug-loaded bone scaffold powders; (**C**) fabrication of the scaffolds via SLS; (**D**) The three orthographic views of the BS-PLLA/PGA scaffolds.

Figure 1B shows the major methods used to prepare composite powders: (a) PLLA and PGA powders were weighed in a ratio of 7:3 and placed in a beaker with anhydrous ethanol. (b) The two powder mixtures were stirred on a magnetic stirrer for 1 h and ultrasonically dispersed for 30 min. (c) The solution containing the powder was transferred to a 50-mL centrifuge tube and centrifuged at 1000 r/min for 5 min, and the supernatant was discarded. The precipitate was dried under vacuum at 37 °C to a constant weight. (d) The BS drug particles were weighed according to their content and added to the aforementioned powder mixture. The contents were stirred in a vortex apparatus for 40 min. (e) Finally, the BS-PLLA/PGA powder for scaffold fabrication was obtained by vacuum drying to a constant weight.

A 3D-printed BS-loaded PLLA/PGA scaffold was fabricated using SLS technology. The SLS system is mainly composed of a CO_2_ laser (SR 10i, ROFIN SINAR Laser GmbH, Hamburg, Germany), a 3D galvanometer scanner (SCANHEAD-300-15D. Century Sunshine Technology Co., Ltd., Beijing, China), and a computer control system. SLS is an additive manufacturing technique wherein 3D scaffolds are fabricated in a layer-by-layer manner based on slice data (section profiles) provided by 3D scaffold models that have been designed (Figure 1C). Firstly, the powders were spread on the forming piston with the aid of powder feeding rollers. Secondly, the spread powder layer was scanned with a laser beam based on the slicing data; that is, the powder particles under the laser scanning area were allowed to sinter together to form a region under laser scanning. Powder delivery pistons were allowed to ascend after one layer was sintered, whereas the forming pistons were allowed to descend according to slice thickness. New layers of powder were subsequently spread and sintered in the same manner until the scaffold was completely prepared. After the un-sintered powders in the scaffold were removed under high-pressure air, the porous scaffold was obtained. The main processing parameters were set as follows: laser power, 2 W; scanning speed, 200 mm/s; scanning space, 0.1 mm; and layer thickness, 0.1 mm.

### 2.2. Extraction and Preparation of BS

The extraction and preparation of BS were carried out by the reflux method [32,33,34]. In this methodology, *Semen strychnine* seeds were powdered and transferred to a round-bottomed flask. The fabrication process of BS was displayed in Figure 1A: (a) The 1/10th amount of lime was stirred with the powder in a round-bottomed flask and wetted with water to cover the powder. (b) The mixed powder was reflux-extracted with benzene solvent for seven times (40 min/time). (c) The benzene extract was collected and condensed to half of its original volume by rotary evaporation. Then 1/10 of the amount of 6% hydrochloric acid was added, and then the white crystals of strychnine hydrochloride were precipitated. (d) Strychnine hydrochloride was filtered, then the extracted acid solution was collected into a beaker, and 1/3 volume of chloroform was added. The pH was adjusted to 12 with ammonia. € A certain amount of sulfuric acid was added to the beaker, and then the BS crude product was precipitated. (f) The crude product was decolorized with activated carbon, washed repeatedly with 95% alcohol, and finally dried to obtain pure BS drug powder. BS crystals were dried at 80 °C under a vacuum. It was obtained as a white crystalline substance and had a higher solubility than brucine.

The extracted BS was analyzed for its composition and purity using HPLC [35,36]. The HPLC conditions were expressed as follows: the mobile phase was water (1% formic acid and 0.3% triethylamine) and methanol in a 75:25 ratio. The Inert-Sustain C18 column (250 mm × 4.6 mm) was adapted to a temperature of 30 °C. The flow rate of the column was set at 1.0 mL/min, and a photo-diode array detector was used at 264 nm. The mixture standards of strychnine and brucine, standard brucine, and extracted samples were dissolved by the mobile phase, and 5 μL of solution was injected into the HPLC instrument, respectively. The components in the extracts were identified by comparing their retention time with that of the standard samples. The standard curve of brucine was established by various concentrations of standard solutions, and the curve was used to calculate the purity of the extracted BS.

### 2.3. Antibacterial Property of BS

*S. aureus* and *E. coli* (standard and antibiotic-resistant strains) were inoculated on a sterile nutrient agar medium and cultured at 37 °C. The bacterial suspensions were prepared with sterile saline solution (0.9% NaCl), and the concentration was adjusted to 10^8^ colony-forming units (CFU)/mL using McFarland standards, after which the stock solutions were diluted to a concentration of 10^6^ CFU/mL before use. Nutrient agar medium was mixed with two kinds of bacterium suspensions (standard *S. aureus* or *E. coli*, concentration: 10^6^ CFU/mL) at a 1:20 ratio. The minimum inhibitory concentration (MIC) was evaluated by the Oxford cup method with different concentrations of BS solution (50 μL). The nutrient broth was used as the control. The cultures were cultivated at 37 °C for 24 h. The experiment was performed three times.

To clarify the minimal bactericidal concentration (MBC) and antibacterial rate, the inhibition time curve was constructed. The bacterial suspension (10^6^ CFU/mL) was diluted to 10^4^ CFU/mL with a gradient of lysogeny broth (LB). Subsequently, the BS solution (400 mg/mL) was diluted to 5, 10, and 20 mg/mL with the diluted bacterial suspension (10^4^ CFU/mL). At the predetermined time points (24, 48, and 72 h), a multimode microplate analyzer (Synergy^TM^, Bio Tek, Winooski, VT, USA) measured the optical density of the suspensions at 600 nm.

The antibacterial rate was expressed as a percentage according to Equation (1) [37]:

Antibacterial rate (%) = 100% × (C − T)/(C − T_0_) (1)

where C is the optical density of the control solution (culture medium with bacteria), T is the optical density of the sample wells, and T_0_ is the optical density of the blank wells (culture medium without bacteria).

The inhibition concentration curve of BS was determined using the turbidimetric method. The bacteria culture was treated with BS (2.5, 5, 10, 20, and 30 mg/mL) and incubated for 20 h at 37 °C. After incubation, 8 μL of the 2,3,5-triphenyltetrazolium chloride dye (TTC. 1-wt%) dye was added to each well, and the plate was incubated for 4 h. Optical density was measured at 600 nm using an enzyme marker, and turbidity was assessed through a glass tube. The antibacterial rate was calculated using Equation (1), and all experiments were performed three times.

### 2.4. Microstructure and Slow-Release Ability of the Scaffold

The morphology of the BS-PLLA/PGA bone scaffolds was characterized by scanning electron microscopy (SEM, Phenom-World BV, Eindhoven, The Netherlands). Elemental analysis of carbon (C), nitrogen (N), and oxygen (O) in the scaffolds was performed using energy-dispersive X-ray spectrometry (EDS, Phenom World BV, Eindhoven, The Netherlands). The bone scaffold powders were fixed on a sample tank for X-ray diffraction analysis (XRD, Shimadzu 6100, Tokyo, Japan) to determine the morphology of the drug present in the scaffolds based on peak patterns and peaks.

Based on the determination of BS release, the release behavior of BS in scaffolds with different BS contents (0%, 1%, 2%, 4%, and 8%) was examined. The scaffold was immersed in a tube containing simulated body fluid (SBF) at pH 7.4 and incubated at 37 °C. At predetermined incubation times (0.5, 1, 2, 4, 6, 8, 12, 22, 34, 46, 56, and 68 h), 1 mL of the scaffold soak solution was aspirated for evaluation, and 1 mL of fresh SBF solution was added. The BS concentration in the aspirated soak solution was measured using HPLC, and the BS release rate from the scaffolds was calculated.

### 2.5. Antibacterial Properties of Drug-Loaded Bone Scaffolds

The antibacterial properties of drug-loaded bone scaffolds were tested using standard strains of *E. coli* and *S. aureus*. All tested stands were sterilized under UV light for 1 h. Bacterial suspensions were diluted to 10^8^ CFU/mL with saline according to the McFarland turbidity standard. Standard strains of *E. coli* and *S. aureus* were diluted to 10^7^ CFU/mL using LB. Bacterial suspensions (10^7^ CFU/mL) were added to test tubes containing drug-loaded bone scaffolds for co-culture, and then the tubes were placed on a rotary shaker. After incubation at 37 °C for a specific time, the scaffolds were removed. Subsequently, 4 μL of 1% TTC dye was added to the bacterial suspension-containing tubes and incubated for 4 h, after which images of the tubes containing the bacterial suspension were captured and directly observed for the color shades. A microplate reader was used to proportionally measure the turbidity of the bacterial suspension solution by determining the optical density at 600 nm. Equation [38], the bacterial inhibition rate was calculated:

Inhibition rate (%) = 100% × (B − A)/B (2)

where B is the optical density of the bacterial suspension incubated with or without the scaffolds, and A is the optical density of the bacterial suspension incubated without scaffolds, respectively. The tests were performed in triplicate.

To determine the interactions between the scaffolds and the cells and to assess the morphology of the bacteria after treatment with drug-loaded bone scaffolds, the scaffolds were analyzed by SEM after co-culture with the bacteria. After incubation at 37 °C for 24 h and 72 h, the scaffolds were removed and gently washed with phosphate-buffered saline (PBS). They were then fixed with 2.5% glutaraldehyde for 4 h, followed by a gentle wash with PBS. A graded series of alcohol was used to dehydrate the scaffolds, after which they were dried at 37 °C for 12 h. In the final step, gold was sprayed on the scaffolds, and they were visualized by SEM analysis.

### 2.6. Bacteriostatic Mechanism

To unravel the antibacterial mechanism of drug-loaded bone scaffolds, the morphological changes in both bacterial strains after the antibacterial effect were analyzed by SEM and transmission electron microscopy (TEM. Phenom-World BV, Eindhoven, The Netherlands). The bacterial suspension (10^5^ CFU/mL) was incubated at 37 °C for 18 h. After removing the medium, the bacteria were resuspended in a nutrient broth medium and treated with BS (30 mg/mL) for 3 h. The bacteria were then harvested by centrifugation and subjected to fixation, staining, dehydration, lyophilization, and finally gold-spraying for SEM analysis. For TEM analysis, embedded blocks were prepared by dehydration, after which they were cut into ultra-thin sections and stained. Finally, the stained sections were observed under a TEM microscope.

### 2.7. Cytocompatibility Assay

A cytocompatibility test was performed on the scaffolds containing MG63 cells (Cell Bank of Type Culture Collection of the Chinese Academy of Sciences, Shanghai, China). The scaffold was tested under in vitro cell culture conditions. Place the sterilized support in a 6-well plate, which contains 1 mL of complete culture medium per hole. The culture medium consists of the minimum necessary culture medium (Gibco, Beijing, China), 10% fetal bovine serum (Gibco, Melbourne, Australia), and 1% penicillin G-streptomycin (Gibco, Beijing, China). Subsequently, the cell suspension of 2 × 10^5^ cells was added to each scaffold and co-cultured in a humidified atmosphere at 37 °C with 5% CO_2_ in the air.

The cytotoxicity of the scaffolds was assessed by nuclear staining with 4′,6-diamidino-2-phenylindole (DAPI. Solarbio, Beijing, China), which was diluted to 0.001 mg/mL with PBS. The scaffolds and cells were co-cultured for specific time points (24, 48, and 72 h). The scaffolds were removed, and cells in a 6-well plate were washed twice with PBS, after which the cells were fixed with paraformaldehyde for 30 min at room temperature. Subsequently, the cells were stained with DAPI and allowed to stand in the dark for 20 min. Finally, the cells were purged twice with PBS and then observed under a fluorescence microscope.

### 2.8. Statistical Analysis

The measurement data were described as mean ± SD. A bilateral *t* test was used to compare the differences between the two groups. Differences among different groups were determined by single factor moment difference analysis (ANOVA) and least significant difference (LSD). All data were processed using GraphPad Prism 9.5.1 software, with Labels *, **, *** and **** representing *p* < 0.05, *p* < 0.01, *p* < 0.001 and **** *p* < 0.0001, respectively.

## 3. Results and Discussion

### 3.1. High-Purity BS Extracted from Semen Strychnine

**Figure 2:**
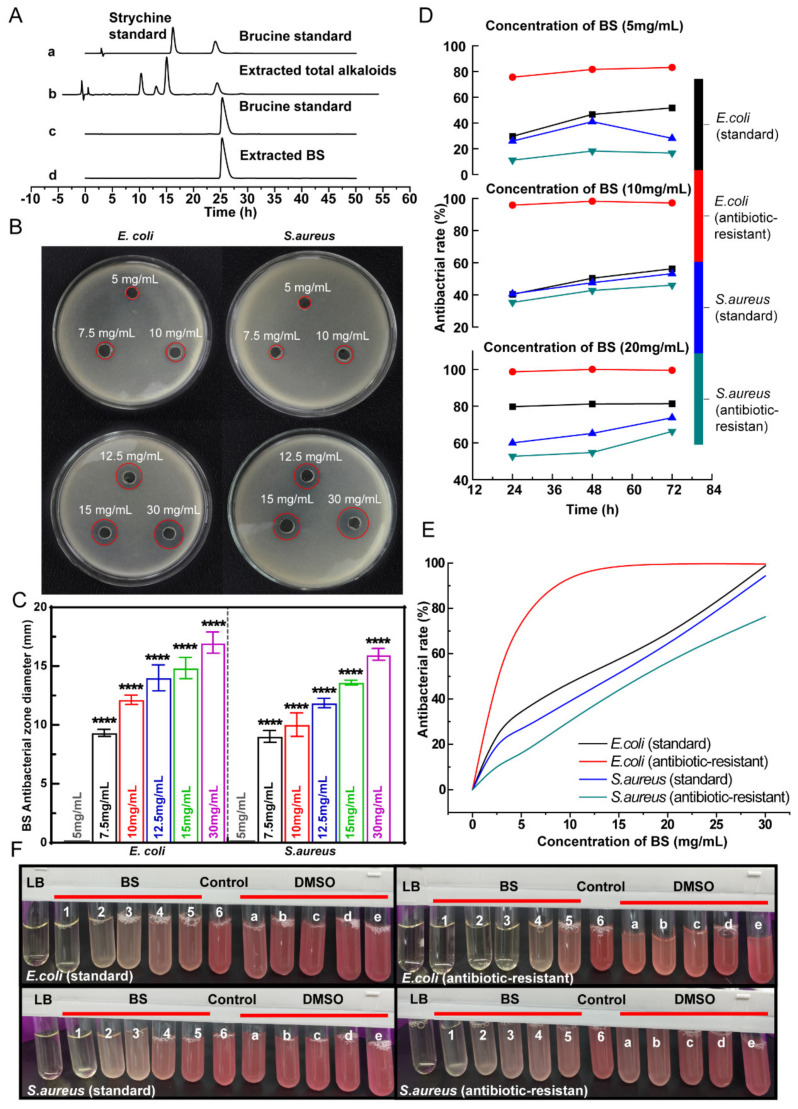
(**A**) HPLC chromatograms. Strychnine and brucine standard mixture representatively (a), total alkaloid extracts (b), brucine standard (c), and extracted BS (d); (**B**) Inhibition zone of BS against standard *E. coli* and *S. aureus* at different concentrations of 5, 7.5, 10, 12.5, 15, and 30 mg/mL; (**C**) The time-courses of BS at 5, 10, or 20 mg/mL against standard and antibiotic-resistant *E. coli* and *S. aureus*; (**D**) BS maximum bactericidal concentration simulation curve; (**E**) The turbidity images of *E. coli* and *S. aureus* at 24 h of incubation. (**F**) Tube LB: no bacteria (blank medium control); Tubes 1–5: BS at 30, 20, 10, 5, and 2.5 mg/mL; Tube 6: bacterial medium control; and Tubes a–e: DMSO controls (corresponding to the concentrations used in BS solutions).

Different extraction methods have different effects on the content and purity of BS obtained from extraction [39,40]. TASS extracts of *semen strychnine* were prepared through reflux extraction, and BS was recovered through crystallization. The purity of BS was evaluated by HPLC analysis (Figure 2). The identification of the ingredients of the extracts was accomplished by comparing the retention time of the samples to the retention time of the standards. The chromatogram of TASS presented four peaks, which indicated that at least four alkaloids were contained in TASS, one of which was attributed to brucine. In crystalized extracts, only one peak indicated that it was BS (Figure 2A(c,d)). The quantification of the extracted BS was performed by the external standardization method, which detected the purity of the extracted BS at 95.3%. The extracted BS was the white crystal, which was an indole alkaloid.

### 3.2. BS Antibacterial Property

The antibacterial activity of BS was tested on two bacterial strains using the Oxford cup assay. The inhibition zone diameters of BS at different concentrations are shown in Figure 2B. The growth of *S. aureus* (standard) and *E. coli* (standard) was inhibited by BS in a concentration-dependent manner. At 5 mg/mL of BS, no inhibition zone was observed, but an inhibition zone appeared at 7.5 mg/mL of BS. As the concentrations increased, the inhibition zones appeared to enlarge. Thus, it was possible to predict that the MIC of BS against *S. aureus* and *E. coli* was both 10 mg/mL. 

Based on the initial results of the Oxford cup assay, the antibacterial effect of BS against *E. coli* and *S. aureus* (a standard and antibiotic-resistant strain) was assessed by turbidimetric and optical density methods. The inhibition time curves of *E. coli* (standard and antibiotic-resistant strains) and *S. aureus* (standard and antibiotic-resistant strains) at different concentrations of BS are shown in Figure 2C. Evidently, BS had an antibacterial effect on standard bacteria and corresponding antibiotic-resistant bacteria in 3 days. There was a highlight that BS (5 mg/mL) has a better antibacterial effect on antibiotic-resistant *E. coli* (83.17%) compared with standard *E. coli* (51.77%) on the 3rd day. Moreover, with the concentration increased to 10 mg/mL, the antibacterial rate of BS against antibiotic-resistant *E. coli* reached 97.22%.

To investigate the antibacterial activity of BS against antibiotic-resistant strains, the inhibition concentration curves of BS were examined. The simulated maximum bactericidal curves of BS against standard and antibiotic-resistant strains of *E. coli* and *S. aureus* are shown in Figure 2D. The simulation curves showed that BS had superior antibacterial activity against *E. coli* antibiotic-resistant strain. The TTC staining results are shown in Figure 2E. The antimicrobial effect was determined by comparing the turbidity and the shade of red of the TTC suspensions in the glass tube. The color and clarity of the solution in the test tube were negatively correlated with the bacterial inhibition. In the TTC staining assay, the lighter the red color of the bacterial suspension and the clearer the transparency, the better the antimicrobial effect. When the BS concentration was from 5 to 30 mg/mL, the bacterial suspensions were brighter as the BS concentration increased. More specifically, at BS concentrations of 10 mg/mL and 20 mg/mL, the resistant *E. coli* suspensions were almost identical to the blank control. These results were consistent with the trend of the modeled curves in Figure 2D, which indicated BS is a new promising candidate drug against antibiotic-resistant bacteria with a long-lasting antibacterial effect.

The above data indicated that BS had bacteriostatic activity against standard and antibiotic-resistant strains of *E. coli* and *S. aureus*. Surprisingly, BS had better bacteriostatic activity for *E. coli* antibiotic-resistant strains than *E. coli* standard strains. Bacterial infection is a huge menace for human health, being one of the major causes of morbidity and mortality [41,42]. Antibiotic resistance has been a persistent issue that causes numerous clinical symptoms [43,44]. In the face of the severe drug-resistant situation, many domestic and international guidelines and studies usually choose appropriate drug combinations to improve the therapeutic effects of these infections [45,46]. However, treatment failure often occurs in clinical practice due to problems such as insufficient dosage administration and serious adverse reactions. Therefore, the extraction of novel antimicrobial drugs from natural plants has become a particularly new approach. The study of BS in the treatment of antibiotic-resistant bacterial infections is still in the preliminary stage, but its remarkable antimicrobial properties and inhibitory effect on antibiotic-resistant strains would make it possible to become a new choice in the future. It is expected that BS could be prepared as a slow and controlled release formulation for local release to reduce the adverse effects and enhance the therapeutic effects.

### 3.3. Physicochemical Properties and Drug Release Behaviors of the Scaffolds

**Figure 3:**
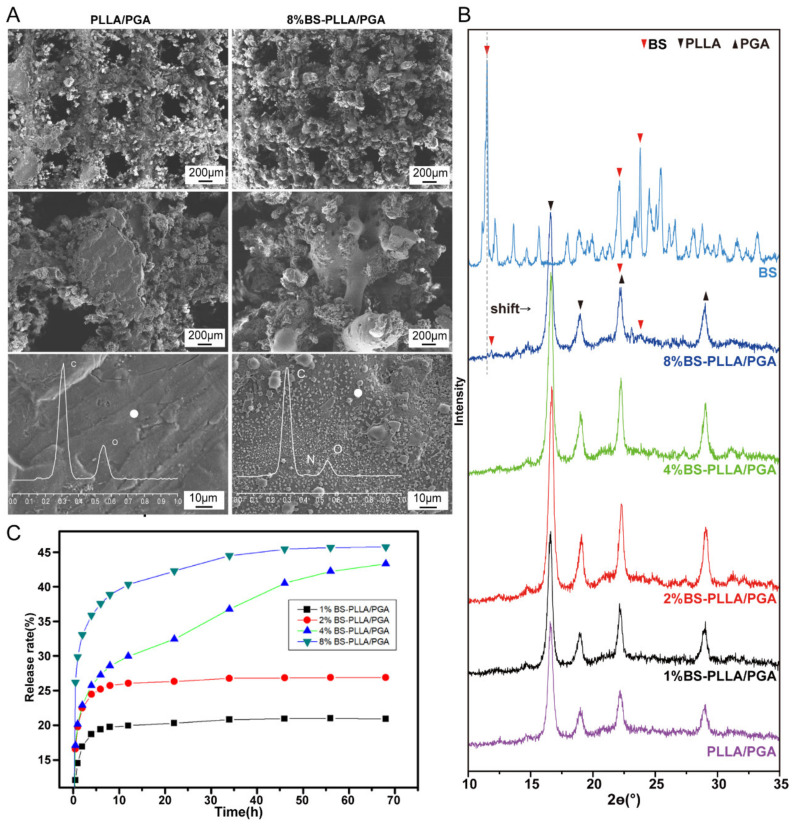
(**A**) The SEM morphology of the scaffolds; (**B**) The XRD patterns; (**C**) Cumulative release curve of BS-PLLA/PGA scaffolds containing different BS contents.

A representative 3D-printed BS-PLLA/PGA scaffold is shown in Figure 1C. The isometric and top views representing the BS-PLLA/PGA scaffold is shown in Figure 1D. The bone scaffold should be mechanically strong enough to withstand stress transfer in the area of the bone defect to provide support for the growth of new bone tissue [47,48,49]. The morphology of the BS-PLLA/PGA bone scaffold system was shown in Figure 3A by SEM analysis. An ordered porous structure of the scaffold with large pores of approximately 500 nm size was exhibited, which was suitable for the inward growth of bone cells and tissues [50]. The micro-pores could facilitate cell adhesion, uptake of biological metabolites, and cell proliferation [51,52]. The internal architecture of the scaffold would directly determine the cellular microenvironment and affect cell growth, signaling, and osteogenic differentiation, depending on the cell type [53]. Cell migration and infiltration within the scaffold were affected by pore size [54,55]. At high magnification, the surface of the PLLA/PGA scaffold was smooth and dense. By contrast, the surface of the BS-PLLA/PGA scaffold was relatively rough, and some particles were distributed in the polymer matrix. Based on EDS results, the presence of an N-elemental peak confirmed that particles belonged to BS, which is due to BS being a nitrogenous compound whereas the polymer matrix does not.

The XRD patterns are shown in Figure 3B. For PLLA/PGA scaffold, four diffraction peaks were detected. The (200)/(110) and (014)/(203) crystal faces of PLLA were detected at two diffraction peaks at 16.56° and 19.00° [56]. The two diffraction peaks at 22.22° and 29.08° are attributed to the (110) and (020) crystal planes of PGA, respectively [57]. Moreover, it was noteworthy that the diffraction angle of BS shifted to 11.48° from 11.84°. Meanwhile, the intensity of the diffraction peak was significantly higher in the low-concentration group compared to the high-concentration drug-loaded scaffold group. These changes indicated the regular and periodic arrangement of PLLA and PGA was disturbed, which was ascribed to the intercalation of BS drug particles into the interlayers of PLLA and PGA. For 4% and 8% BS-PLLA/PGA scaffolds, the diffraction peaks at 16.6° and 19.0° of BS were detected, but the other scaffolds were not. It may be that the content of BS was so low that it exceeded the detectability of the XRD instrument. The above results showed that the crystal structure and chemical structure of BS drug particles did not change after 3D printing with the matrix material powder.

The release behavior of BS in scaffolds with different contents was investigated by detecting the amount of BS released. As the drug loading increased, so did the amount of drug released from the scaffold. As shown in Figure 3C, there are two phases of drug release behavior. In the first phase, the release rate of drug-loaded bone scaffolds was rapid within 10 h. While in the second phase, the rate of release was relatively flat after 12 h. This release behavior was mainly due to the unique layered laser scanning method of SLS used to prepare the scaffold, which caused the drug particles attached to the surface of the scaffold to dissolve and be released quickly [58,59]. While the BS drug particles sintered into the matrix material by laser were slowly released due to the degradation of the scaffold. The long-term sustained release of BS was realized by the SLS preparation technology of drug-loaded bone scaffolds. In the high-concentration group (4%, 8% BS-PLLA/PGA scaffolds), more drugs attached to the surface were dissolved, which made SBF enter the interior of the scaffold easier, resulting in faster matrix-material degradation and correspondingly more drug release. The rapid release of the drug in the first phase could allow BS to reach an effective concentration quickly enough to play a therapeutic role. The subsequent slow and sustained release maintained the efficacy of BS while avoiding its toxic effects.

### 3.4. Antibacterial Properties of the Scaffolds

**Figure 4:**
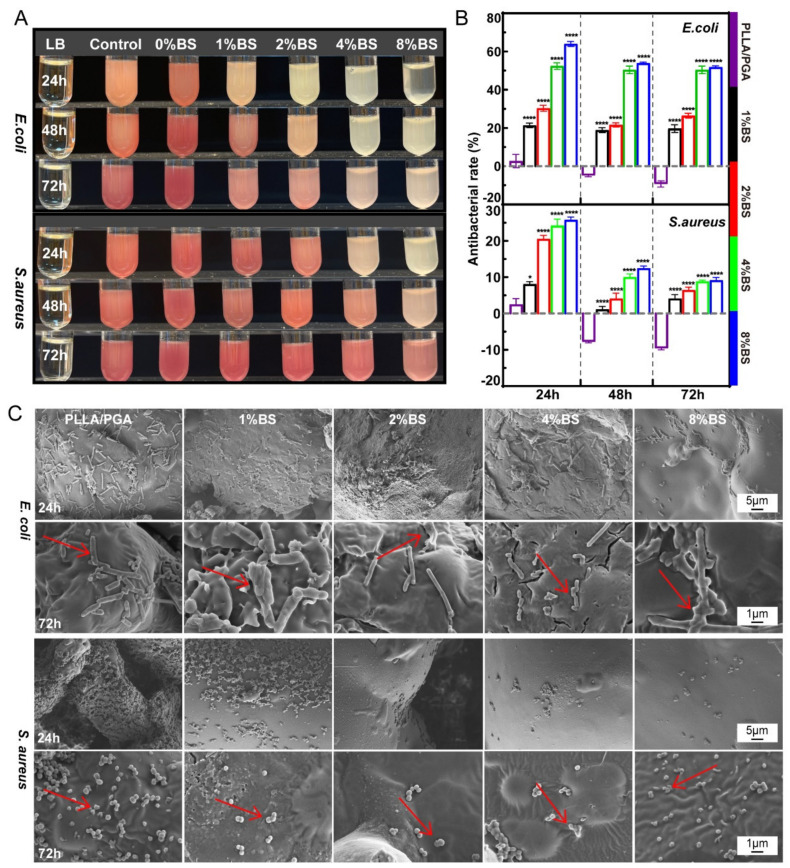
(**A**) The turbidity image of *E. coli* and *S. aureus* in co-culture with the scaffolds after 24 h, 48 h, and 72 h; (**B**) Antibacterial rates of drug-loaded scaffolds with different concentrations of BS against standard *E. coli* and *S. aureus* at 24 h, 48 h, and 72 h; (**C**) The adhesion morphology of standard *E. coli* and *S. aureus* on the scaffolds of different concentrations at 24 h and 72 h.

Figure 4A showed that the scaffolds were incubated in the *E. coli* and *S. aureus* suspensions for 24, 48, and 72 h. The bacteria were stained with TTC to prevent the degradation powder of the scaffold from influencing the observation. As the BS content increased in the different scaffold groups, the color of the suspension gradually became shallower. Interestingly, after co-culture with the PLLA/PGA scaffold, the turbidity of the bacterial suspension became turbid instead of decreasing. By contrast, the solution in the 8% BS-PLLA/PGA scaffold group was significantly lighter in color. This indicated that the BS killed a lot of bacteria. For the low concentration BS-PLLA/PGA scaffold, the bacterial suspension turbidity did not change significantly, indicating relatively weak antibacterial activity. 

The antibacterial rates were calculated using the absorbance of bacterial suspensions (Figure 4B). During co-culture, BS-PLLA/PGA scaffolds had a better antibacterial effect against standard *E. coli* compared with standard *S. aureus*. The antimicrobial activity of the scaffolds was consistent with the initial examination of BS monotherapy. The 4% and 8% BS-PLLA/PGA scaffolds showed 52.39% and 64.00% inhibition of standard *E. coli* within 24 h, respectively. Over the next 2 days, the inhibition of standard *E. coli* remained at about 50%. This suggests that drug-loaded bone scaffolds have successfully achieved continuous and effective antimicrobial action. Interestingly, after co-culture with the PLLA/PGA scaffold, the turbidity of the bacterial suspension became turbid instead of clear. PLLA/PGA scaffolds had a negative antibacterial rate. It is presumed that the structural characteristics of the scaffold system were more conducive to bacterial adhesion [60,61,62]. The porous structure of the scaffold provided an attachable growth space for bacterial growth [63]. This indicates that the bone scaffold system was not effective in preventing infection during bone transplantation. 

In the grafting of bone scaffolds, operations often fail due to infection. Therefore, the development of a bone scaffold with a bacteriostatic effect will greatly reduce the risk of surgical infection and improve the success rate of bone grafting. The BS-PLLA/PGA bone scaffold system would be expected to remedy this deficiency. The BS drug-loaded bone scaffold could achieve a local release function and a long-term effective antimicrobial action, thus improving its utilization efficiency. Afterwards, the local release property of BS could significantly reduce the side effects caused by systemic administration and improve the safety of the treatment process. The materials for the construction of bone scaffolds should have good mechanical properties and biocompatibility, so as to promote the regeneration and repair of bone cells and tissues. Based on this, as a novel therapeutic tool, drug-loaded antimicrobial bone scaffolds would have significant advantages in local infection control and bone tissue repair.

### 3.5. Antibacterial Mechanism

The adhesion of standard *E. coli* and *S. aureus* co-cultured in different concentrations of drug-loaded scaffolds for 24 and 72 h is shown in Figure 4C. In the PLLA/PGA scaffold, *E. coli* and *S. aureus* appeared rod-shaped and rounded with an intact and smooth appearance, typical of normal *E. coli* and *S. aureus*, respectively [64]. *E. coli* appeared as a long chain, linked from the beginning to the end, and *S. aureus* appeared as stacked aggregates. Most of the surface of the scaffold was covered by bacteria. By contrast, the number of bacteria on the high-concentration scaffold was significantly reduced. More importantly, their cellular structures were severely damaged. 

**Figure 5:**
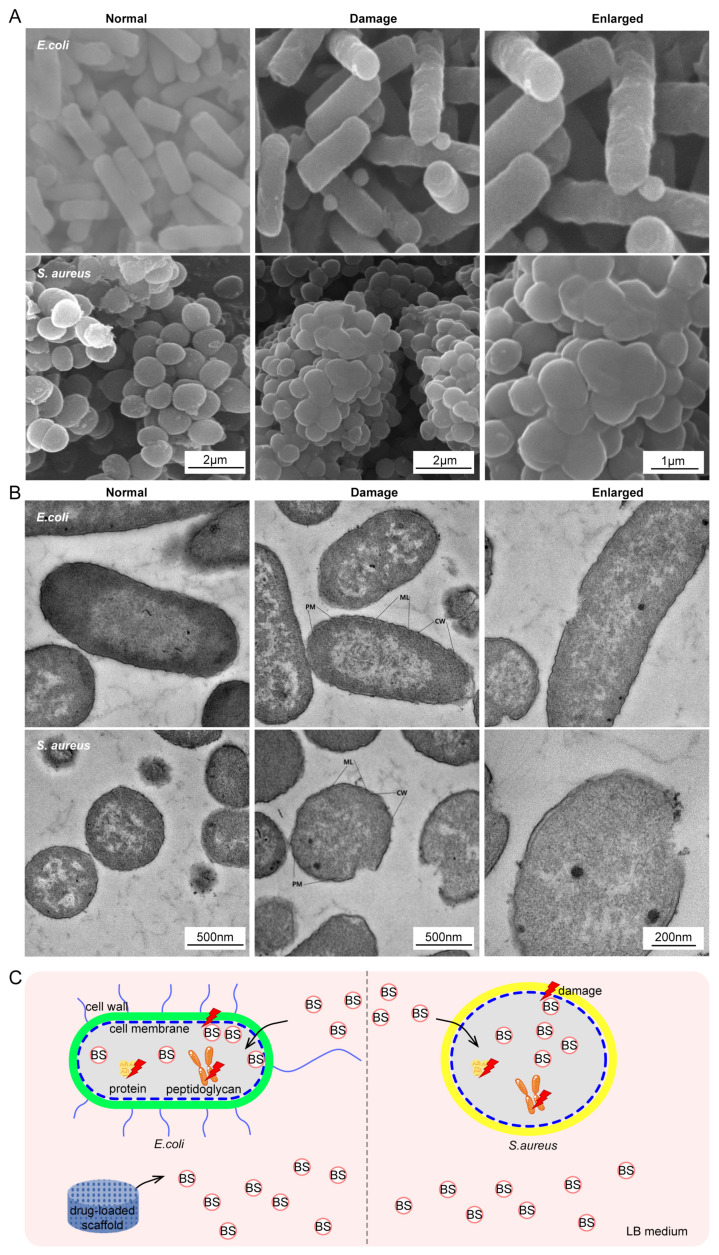
(**A**,**B**) SEM and TEM images of standard *E. coli* and *S. aureus* treated by BS for 3 h; (**C**) Schematic diagram of possible antibacterial mechanisms of the drug-loaded scaffold.

Figure 5 shows the internal and external morphologies of the bacteria as observed using SEM and TEM, respectively. The surface of the bacteria had a distorted and contracted appearance, together with the rupture and collapse of the bacterial membrane. *E. coli* strains were obviously edema, demonstrating local dissolution of the intracellular matrix [65]. The protein mucosal layer (ML) was thin and disappeared locally. The cell wall (CW) appeared wrinkled and broken. The plasma membrane (PM) was slightly depressed inward, with plasma wall separation, membrane breakage, and dissolution, and the periplasmic interval was blurred. Meanwhile, *S. aureus* strains had slight edema with a slightly shallow intracellular matrix. ML was thin, with a small local loss. CW crinkled and broke locally. PM was locally damaged with a slight wrinkle and no significant plasma wall separation. 

In summary, the antibacterial effect of the drug-loaded bone scaffold system was mainly achieved by the drug-killing effect of BS. A possible synergistic antibacterial mechanism of the drug-loaded bone scaffold system is shown in Figure 5C. BS was gradually released with the degradation of the scaffolds. Then, it entered bacterial cells and disturbed the synthesis of peptidoglycan and protein, causing the bacteria to produce structural proteins and enzymes, which inhibited the growth of bacteria.

### 3.6. Cytocompatibility

**Figure 6:**
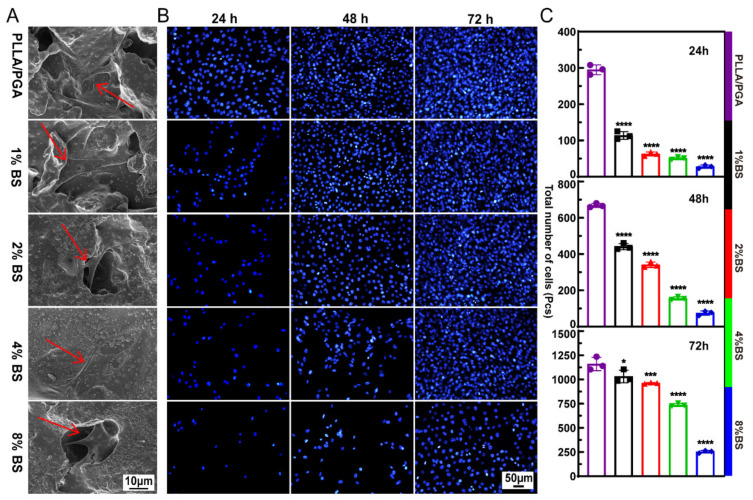
(**A**) The adhesion morphology of MG63 cells in co-culture with the scaffolds after 3 days; (**B**) Fluorescence microscope images of MG63 cells co-cultured with the scaffold for 24, 48, and 72 days (nuclei shown as blue dots); (**C**) The total number of MG63 cells after co-culture with the scaffolds.

SEM analysis was used to observe the adhesion morphology of MG63 cells cultured for 3 days on the scaffolds (Figure 6A). All MG63 cells were present in close contact and adhered to the porous structure of the scaffold [66]. As for the morphology of the cells, they are elongated and plump, with filamentous pseudopods indicating good cell attachment [67,68,69]. As shown in Figure 6B, cell fluorescence staining was performed after 24, 48, and 72 h of co-culture. Cells in co-culture with the scaffold show well-distributed morphology. For the high-concentration drug-loaded bone scaffold group, the number of cells was significantly different from the other scaffold groups after co-culture for 24 to 48 h. This may be attributed to the fact that BS acts as an inhibitor of cancer cells, resulting in the inhibition of the growth of MG63 cells, affecting their growth in the first 48 h [70]. And the number of cells on the scaffold gradually increased with the increase in culture time. This again demonstrated their excellent cytocompatibility [71,72,73,74].

## 4. Conclusions and Future Prospects

In this study, well-water-soluble BS was successfully extracted and prepared using the reflux method, which had a purity of up to 95.3%. BS, as a natural medicine extract, had good antibacterial activity. The results showed that Gram-positive (*S. aureus*) and Gram-negative (*E. coli*) were markedly inhibited by BS, especially for clinical antibiotic-resistance *E. coli* compared to standard bacteria *E. coli*. These findings indicated that BS has the potential to become a novel antibiotic substance and relieve the problem of antibiotic resistance. BS as a natural herbal extract is characterized by high toxicity and a narrow therapeutic window, while its treatment effect is remarkable. Within the range of safe dosages, the therapeutic efficacy of the drug is enhanced with the increase in dosage, but its adverse effects also increase significantly. In future studies, we would modify the dosage form of BS drugs to evaluate their safety and efficacy with the expectation of maximizing efficacy within a safe range. In this study, we conducted a preliminary investigation on the antimicrobial activity and mechanism of BS. We will further study the inhibitory activity of BS against drug-resistant strains and its mechanism of action.

Based on the toxicity problem and satisfactory antibacterial activity of BS, the BS-PLLA/PGA slow-release bone scaffold system was constructed using SLS technology, which would achieve local drug release to reduce toxicity and play a good antibacterial role. The bone scaffold slow-release system had a tight, porous structure and showed effective slow-release of BS. Bacteriostatic tests further validated that the scaffold had sustained and effective bactericidal action. We also explored the mechanism of the antimicrobial property, which revealed that the antimicrobial activity might be attributed to the destructive effect of BS on bacterial CW. Moreover, this scaffold system exhibited good cytocompatibility without affecting cell adhesion or viability. In future studies, we will continue to explore whether this drug-loaded scaffold system is a good material for bone support and anti-infection when it is implanted in animals. We expect that the drug-loaded bone scaffold system could be further optimized. BS would be prepared into microsphere form to improve the drug-loaded capacity of the scaffold, and biomaterials such as chitosan would be added to the bone scaffold system to improve its biocompatibility and induce osteogenesis. It makes the scaffold have better application in bone tissue engineering research.

## Data Availability

The dataset available on request from the authors. The raw data supporting the conclusions of this article will be made available by the authors on request.
